# Computational Characterization of Mechanical, Hemodynamic, and Surface Interaction Conditions: Role of Protein Adsorption on the Regenerative Response of TEVGs

**DOI:** 10.3390/ijms23031130

**Published:** 2022-01-20

**Authors:** Alejandra Riveros, Andres J. Garcia-Brand, Maria A. Rodriguez-Soto, Nestor Sandoval, Carolina Muñoz-Camargo, Juan C. Cruz, Juan C. Briceño

**Affiliations:** 1 Department of Biomedical Engineering, Universidad de los Andes, Bogotá 11711, Colombia; aj.garcia14@uniandes.edu.co (A.J.G.-B.); ma.rodriguezs1@uniandes.edu.co (M.A.R.-S.); c.munoz2016@uniandes.edu.co (C.M.-C.); 2 Department of Congenital Heart Disease and Cardiovascular Surgery, Fundación Cardio Infantil Instituto de Cardiología, Bogotá 111711, Colombia; nsandoval@cardioinfantil.org; 3 Department of Research, Fundación Cardio Infantil Instituto de Cardiología, Bogotá 111711, Colombia

**Keywords:** tissue engineering vascular grafts (TEVG), protein adsorption, fibrinogen, albumin, two-way FSI, CFD, multiphysics

## Abstract

Currently available small diameter vascular grafts (<6 mm) present several long-term limitations, which has prevented their full clinical implementation. Computational modeling and simulation emerge as tools to study and optimize the rational design of small diameter tissue engineered vascular grafts (TEVG). This study aims to model the correlation between mechanical-hemodynamic-biochemical variables on protein adsorption over TEVG and their regenerative potential. To understand mechanical-hemodynamic variables, two-way Fluid-Structure Interaction (FSI) computational models of novel TEVGs were developed in ANSYS Fluent 2019R3^®^ and ANSYS Transient Structural^®^ software. Experimental pulsatile pressure was included as an UDF into the models. TEVG mechanical properties were obtained from tensile strength tests, under the ISO7198:2016, for novel TEVGs. Subsequently, a kinetic model, linked to previously obtained velocity profiles, of the protein-surface interaction between albumin and fibrinogen, and the intima layer of the TEVGs, was implemented in COMSOL Multiphysics 5.3^®^. TEVG wall properties appear critical to understand flow and protein adsorption under hemodynamic stimuli. In addition, the kinetic model under flow conditions revealed that size and concentration are the main parameters to trigger protein adsorption on TEVGs. The computational models provide a robust platform to study multiparametrically the performance of TEVGs in terms of protein adsorption and their regenerative potential.

## 1. Introduction

Currently available small-diameter vascular grafts (<6 mm) exhibit several long-term limitations, related to their high thrombogenicity limiting their clinical implementation [1,2,3,4,5,6,7]. The development of novel small-diameter vascular grafts could be then addressed by tailoring the regenerative profile of the biomaterials tuning the adsorption of proteins to avoid thrombosis due to platelet adhesion and activation [8].

Cell-surface interactions seems to be regulated by the bioactive properties of the graft surface (e.g., hydrophobicity and charge) and then, a strategy to promote cell adhesion is therefore improving the availability of bioactive sites at the graft lumen to control the orientation and conformation of the adsorbed proteins [9]. Albumin and fibrinogen have been reported to be the predominant proteins to be adsorbed on vascular graft surfaces [10]. In particular, fibrinogen adsorption has been correlated with promoting platelet and monocyte/macrophage adhesion, thereby leading to blood clotting [11], while albumin-coated surfaces seems to reduce leukocyte interaction, mitigating the inflammatory response [10,12].

Studying the dynamics between blood flow, protein adsorption and cell adhesion are key to understand the tissue engineered vascular grafts (TEVGs) outcomes. Multiphysics modeling and simulation that couple Computational Fluid Dynamics (CFD) to conservation of species and even structural mechanics equations has emerged as a powerful tool for a comprehensive understanding of the underlying mechanisms governing blood-biomaterial interactions [13,14,15,16]. Through this approach, it is possible to analyze the interplay of multiple parameters without the expenses of experimental procedures.

Besides recent computational approaches dedicated to better understand the mechanistic behind protein adsorption on biomaterials surfaces intended for the manufacture of TEVGs, key mechanical, hemodynamical and surface biochemical parameters have been evaluated mostly independently, which represents a major limitation specially when it comes to searching for a reliable performance under physiological conditions [10,17,18,19,20,21]. For that purpose, the coupling CFD, Finite Element Methods (FEM) and multiphysics models represents an attractive route to describe much more accurately the protein dynamics when a biomaterial is exposed to biological fluids. This approach seems appropriate to overcome the lack of information to describe in sufficient detail the microenvironment of phenomena occurring at the microscale such as protein adsorption where fluid dynamics and mechanical stimulation interplay as key regulators. Accordingly, here we propose a multiphysics model of the interplay of mechanical, hemodynamic and biochemical variables to study the mechanisms of protein adsorption [10,17,18,19,20,22,23] on TEVGs to provide a robust basis for their rational design.

## 2. Results

### 2.1. Fluid Model

**Figure 1:**
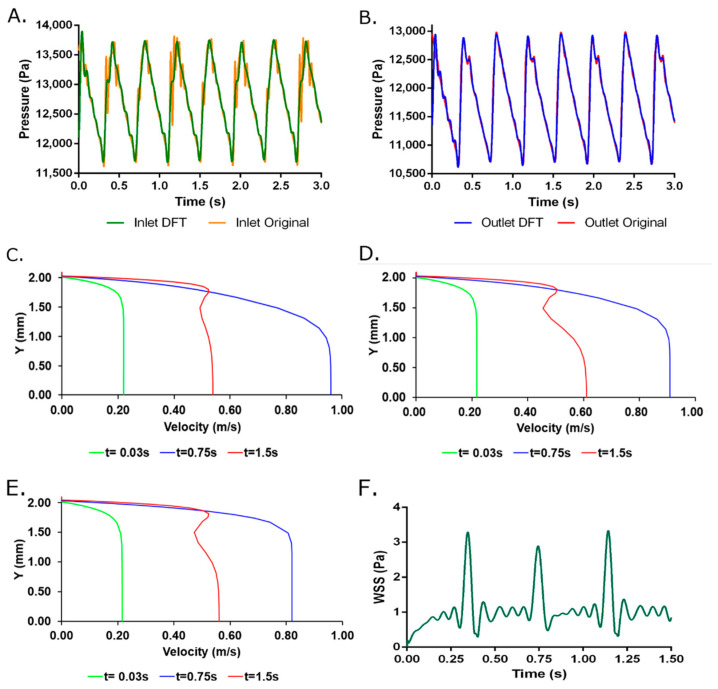
(**A**) Inlet and (**B**) Outlet DFT pressure reconstruction using 125 harmonics compared to experimental data. Velocity profiles for two-way FSI model at (**C**) z = 1 cm (**D**) z = 3 cm and (**E**) z = 5 cm from the inlet face for three selected times. (**F**) Wall Shear Stress obtained from the two-way FSI model. Additional time steps are available in Supplementary Material Animation S1.

Figure 1A,B shows inlet and outlet DFT pressure reconstruction compared to experimental data, which indicates a physiological flow regime imposed on the simulations. A cyclic and pulsatile flow behavior was observed with velocity profiles (Figure 1C–E) fully developed along the geometry at an early time step of t = 0.03 s. These results indicate gradient pressure predominant over inertial forces that are confirmed by the parabolic velocity profile. A behavior that is maintained at an intermediate time of t= 0.75 s for three probe points at x = 0, x = L/2 and x = L where maximum velocities were spatially dependent. However, near to the final simulation time (t = 1.5 s), the velocity profiles exhibit an “s”-shape indicating an inflection point inside the fluid that can be attributed to the prevalence of the inertial forces. Moreover, the average sum of inlet and outlet flow rates of −1.18 × 10^−9^ m^3^/s corroborates the assumption of steady-state of the modeled system.

### 2.2. Two-Way FSI Model

**Figure 2:**
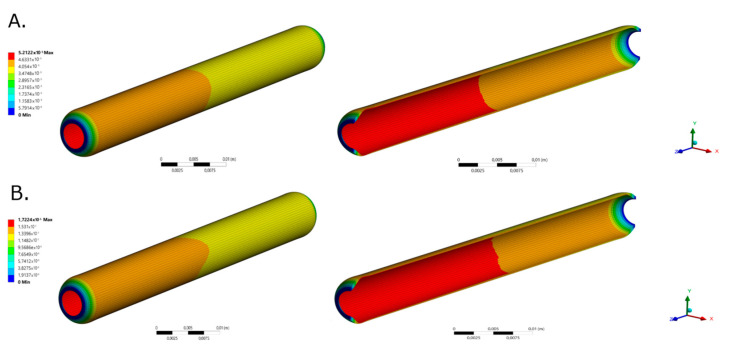
TEVG total deformation for selected time steps for (**A**). time = 0.189 s (Pmax) and (**B**). t = 0.945 s (Pmin), respectively.

**Figure 3:**
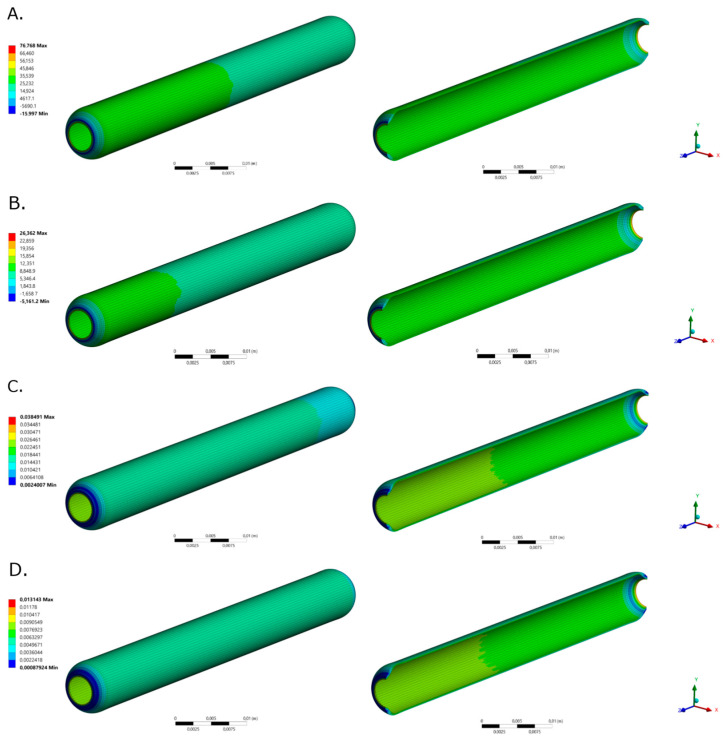
TEVG maximum principal stresses for (**A**). time = 0.189 s (Pmax) and (**B**). t = 0.945 s (Pmin), respectively. TEVG maximum principal elastic strain for (**C**). time = 0.189 s (Pmax) and (**D**). t = 0.945 s (Pmin), respectively.

Figure 2 and Figure 3 shows the total deformation, total principal stresses, and total elastic strain as result of the pulsatile pressure for two-time steps. Additional time steps can be found in Supplementary Material Animation S1. The total maximum stress (0.084 MPa) calculated by the simulation was 5.48 times below the ultimate tensile strength reported for native porcine arteries (0.49 MPa) [24]. The wall shear stresses (Figure 1F) undergo common values for small diameter arteries (0.1 Pa to 5 Pa) that could be associated with optimal values for cell mechanotrasduction [8,25,26] and supported by the maximum elastic strain (4.18%) found lower than the ultimate strain of carotid porcine arteries (180%) [24]. Moreover, these results strongly indicates that the decellularized based TEVG responds dynamically to the physiologically-like operational conditions and do not represent riskiness of mechanical failure due to physiological performance.

### 2.3. Kinetic Model

**Figure 4:**
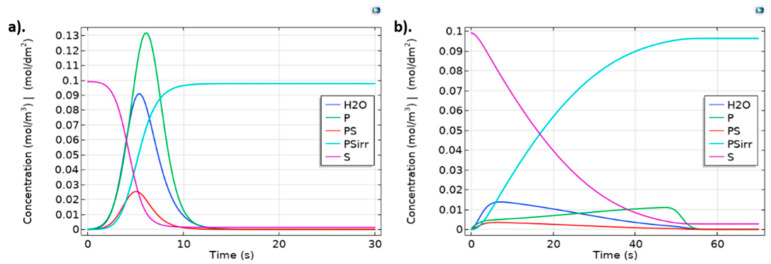
Protein adsorption dynamics for (**a**) Albumin and (**b**) Fibrinogen for *k_r_* = 1, *k_f_* = 9 and *k_i_* = 1. Surface concentrations are scaled (1 × 10^4^ times) to allow comparison with bulk species. Albumin was injected as a pulse lasting for 1.0 s. Fibrinogen was injected in fifty pulses, each one lasting 1.0 s to determine saturation time on geometry the independent model.

From the kinetic reaction analysis (Figure 4), fibrinogen adsorption starts about 2 s earlier than albumin absorption. This kinetic behavior could be explained by the greater affinity of fibrinogen with hydrophobic surfaces as is reported to be the luminal layer of decellularized-based TEVGs [27], contrary to the behavior of albumin, which its aminoacidic structural conformation confers a predominant affinity with hydrophilic surfaces. Then, this result strongly suggests the importance of promoting albumin adsorption over fibrinogen to control the thrombogenic and inflammatory response of the TEVG. Hydrophilic coating has been reported to promote albumin adsorption over fibrinogen adsorption [9].

### 2.4. Two-Way FSI and Protein Adsorption Models Coupling

**Figure 5:**
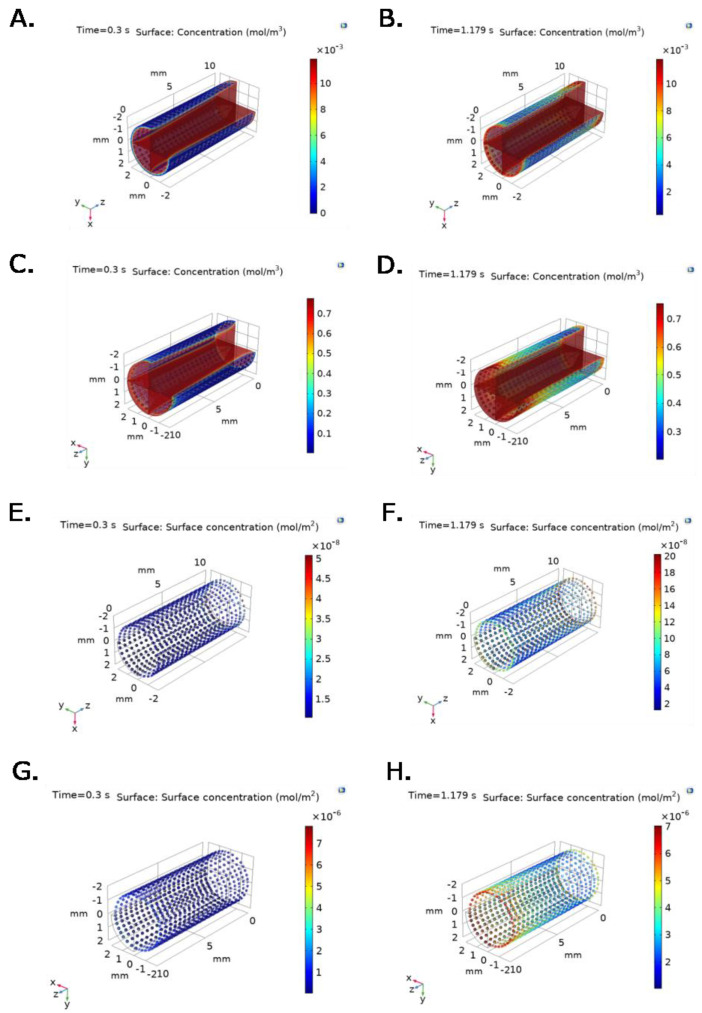
Bulk protein concentration for (**A**,**B**) fibrinogen and (**C**,**D**) albumin at selected time steps for time = 0.3 s (Pmin) and t = 1.179 s (Pmax), respectively, under inlet experimental pressure and constant outlet velocity. P∙S complex concentration for (**E**,**F**) fibrinogen and (**G**,**H**) albumin at selected time steps for time = 0.3 s (Pmin) and t = 1.179 s (Pmax), respectively, under inlet experimental pressure and constant outlet velocity.

Figure 5 confirms that bulk protein concentration is primarily time and spatial dependent. At t = 0.3 s, a maximum TEVG surface fibrinogen concentration of 3 × 10^−3^ mol/m^3^ with an average protein concentration of 1 × 10^−3^ mol/m^3^ is reached for the constant outlet velocity model (Figure 5A). At t = 0.179 s, a maximum TEVG surface fibrinogen concentration of 10 × 10^−3^ mol/m^3^ with an average protein concentration of 5 × 10^−3^ mol/m^3^ is reached for the constant outlet velocity model (Figure 5B).

Figure 5C shows that at t = 0.3 s, albumin concentration reaches a maximum on the TEVG surface of 0.3 mol/m^3^ with an average protein concentration of 0.15 mol/m^3^. Figure 5D shows that at t = 0.179 s, albumin concentration reaches a maximum on the TEVG surface of 0.7 mol/m^3^ with an average protein concentration of about 0.5 mol/m^3^ for the constant outlet velocity model.

Figure 5E–H show that P∙S complex concentrations for albumin and fibrinogen over the TEVG lumen are also time and spatial dependent under experimental pressure at inlet, and constant velocity at outlet of 0.047 m/s. As expected, the active sites availability decreases with time and position, while Surface-Protein complexes concentration increases and levels off after reaching saturation.

**Figure 6:**
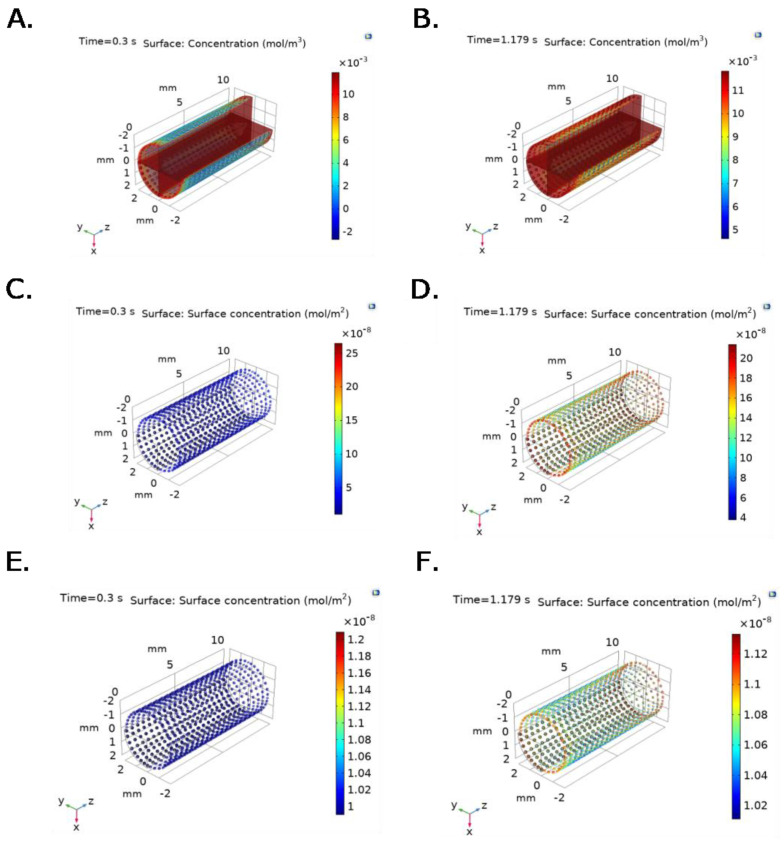
Bulk protein concentration for fibrinogen at selected time steps: (**A**) t = 0.3 s (Pmin) and (**B**) 1.179 s (Pmax). P∙S complex concentration for fibrinogen at selected time steps: (**C**) t = 0.3 s (Pmin) and (**D**) 1.179 s (Pmax). Irreversible P∙S complex concentration for fibrinogen at selected time steps: (**E**) t = 0.3 s (Pmin) and (**F**) 1.179 s (Pmax). Boundary conditions were set as inlet experimental pressure and outlet V(x,t) function imported from the ANSYS Two way-FSI model.

**Figure 7:**
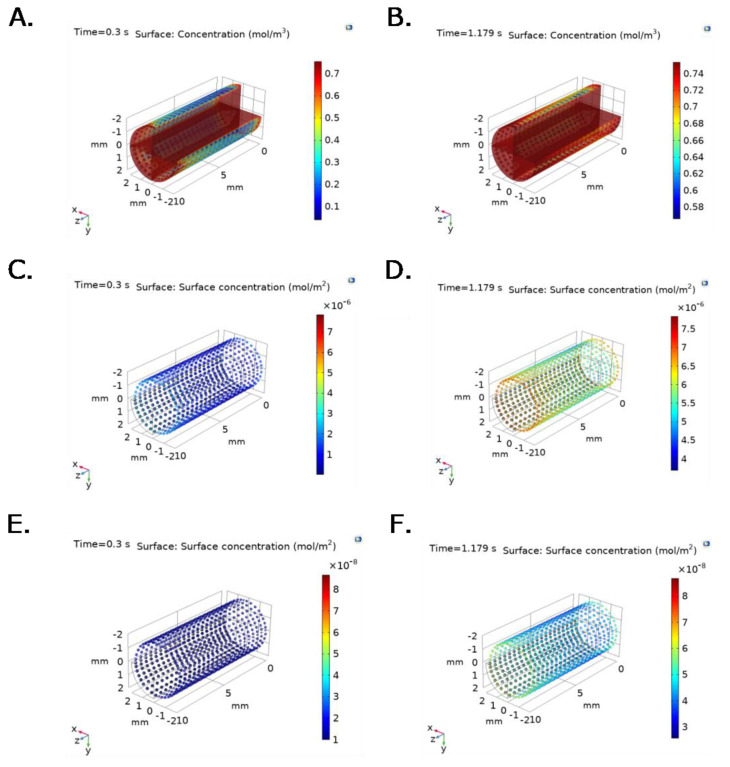
Bulk protein concentration for albumin at selected time steps: (**A**) t = 0.3 s (Pmin) and (**B**) 1.179 s (Pmax). P∙S complex concentration for albumin at selected time steps: (**C**) t = 0.3 s (Pmin) and (**D**) 1.179 s (Pmax). Irreversible P∙S complex concentration for albumin at selected time steps: (**E**) t = 0.3 s (Pmin) and (**F**) 1.179 s (Pmax). Boundary conditions were set as inlet experimental pressure and outlet V(x,t) function imported from the ANSYS Two way-FSI model.

Figure 6 and Figure 7 show time and spatial dependent bulk protein and P∙S complex concentration for albumin and fibrinogen over the TEVG lumen, under experimental pressure at the inlet, and v(x,t) function imported from the ANSYS Two way-FSI model. Active sites concentration decreases with time and position, while Surface-Protein complexes concentration increases and levels off after reaching saturation (Figure 6C–F and Figure 7C–F). At t = 0.3 s, bulk protein concentration, for both fibrinogen and albumin, increases until the protein reacts with active sites, which is evidenced by lower concentrations close to active sites (Figure 6A,B and Figure 7A,B).

Differences in spatial and temporal concentrations under constant velocity compared to v(x,t) condition at the outlet are evidenced in Figure 6, where faster fibrinogen reaction with active sites is observed across the geometry under conditions that approach those observed physiologically (time and spatial dependent velocity function).

A similar behavior is observed in the albumin model (Figure 6) since higher concentration values are reached for the same time interval in the changing outlet velocity model when compared with the constant outlet velocity model presented in Figure 5.

For the changing outlet velocity model, a maximum TEVG surface concentration for fibrinogen of 7 × 10^−3^ mol/m^3^, with an average concentration of 3 × 10^−3^ mol/m^3^, is reached at t = 0.3 s (Figure 6A). At t = 1.179 s, a maximum TEVG surface concentration of 12 × 10^−3^ mol/m^3^ is reached (Figure 6B). A maximum concentration of 0.012 mol/m^3^ was obtained for fibrinogen at t = 0.8 s.

At t = 0.3 s, the maximum TEVG surface albumin concentration is 0.6 mol/m^3^ with an average concentration of 0.3 mol/m^3^ for the changing outlet velocity model (Figure 7A). At t = 1.179 s, a maximum TEVG surface albumin concentration of 0.74 mol/m^3^ with an average protein concentration of 0.68 mol/m^3^ is reached (Figure 7B). Albumin reached a maximum concentration of 0.753 mol/m^3^ at t = 1.39 s.

The experimental validation corroborates the greater agreement of the simulation results obtained with the two-way FSI model compared to previous rigid wall models [16,28]. This was evidenced by a reduction of 36.48% in the error obtained when comparing average computational flow rate of 25-time steps and the experimental flow rate provided by the pump (3.33 × 10^−7^ m^3^/s). Moreover, the average sum of inlet and outlet flow rates of −1.18 × 10^−9^ m^3^/s confirms the assumption of steady-state for the modeled system.

## 3. Discussion

Our two-way FSI model coupled to a multiphysic kinetic model aims to simulate albumin and fibrinogen adsorption on TEVG’s intima surface under physiological-like pulsatile flow conditions with the objective of establishing a correlation between mechanical-hemodynamic-biochemical variables on protein adsorption on TEVGs and their possible impact on the underlying regeneration processes. The model might be useful for estimating the performance of novel TEVGs under physiological flow conditions and its effect on critical events that occur at the molecular levels such as protein adsorption, which is thought to be directly responsible for modulating the subsequent cell adhesion and the TEVG’s overall regenerative response.

Our main findings seem to be accordance with previously reported models for protein adsorption and flow mediated chemical species transport [29,30,31]. Fibrinogen is adsorbed faster than albumin, showing a saturation time of 0.8 s and a maximum concentration of 0.012 mol/m^3^ compared with a saturation time of 1.39 s and a maximum concentration of 0.753 mol/m^3^ for albumin.

In fact, our multiphysics fully coupled model indicates similar time-dependent surface saturation levels compared with those reported by Manzi et al. [32] as evidenced by albumin saturation after about 1.5 s. Results reported by Richert, et al. [33] show that fibrinogen adsorption saturation time occurs around 1 s. Altogether, these results suggest that protein adsorption kinetics for short times can be modeled with relatively high accuracy by coupling a mutiphysic model with a two-way FSI model. This prediction capacity is important because most relevant protein adsorption events defining success or failure occur during the first seconds after blood interaction under pulsatile flow.

Although albumin has a higher concentration than fibrinogen in a typical physiological-like environment (3.5–4.5 g/dL vs. 150–400 mg/dL), the faster fibrinogen adsorption could be explained by the smaller fibrinogen size and the particle size-dependent flow distributions in hemodynamic conditions, where smaller particles locate nearer the vascular wall [34]. Furthermore, adsorbed fibrinogen in the luminal surfaces of blood vessels has been shown to interact with the glycoprotein VI in platelets promoting platelet activation and inducing thrombogenesis [35]. Thus, the need for a surface treatment for preferential albumin adsorption is corroborated by our model to dismiss the thrombogenic plausibility of a designed graft [10,36], especially in small-diameter TEVGs. Consequently, one of the current strategies is tuning the surface properties of the TEVGs including coatings with macromolecules and drugs to significantly modify biomaterials towards hydrophilic surfaces.

Albumin/fibrinogen ration adsorption in different surfaces is key to understanding the thrombogenicity of the biomaterial. In this sense, a high albumin/fibrinogen ratio is correlated with a low platelet adhesion [37]. This effect depends on the hydrogen content of the surface, given that fibrinogen is more hydrophilic than albumin due to its functional groups and 3D configuration, and the albumin/fibrinogen adsorption ratio will decrease on hydrophilic surfaces [9,37,38,39]. Those events are required for irreversible-like albumin adsorption (i.e., *k_f_* < *k_r_* and *k_f_* > *k_r_*) facilitating the rapidly saturation of the reaction sites with an asymptotic behavior for the concentration of the irreversible ($\bar{P}\cdot \bar{S}$) complexes under pulsatile flow conditions that tend to remove reversible protein-surface complexes [10,30].

Additionally, our protein adsorption model is significantly sensitive to the flow complexity and the predominance of inertial forces. Hence, a wave-like time dependent concentration gradient across the tubular geometry provides a suitable approach to understand the flow and the interplay of biochemical variables guiding protein adsorption on TEVGs. Moreover, the model sensitivity to both flow and biochemical conditions validate the presence of intricate relationships among the proposed biochemical, mechanical and hemodynamics variables as experimentally demonstrated by previous studies [40,41].

For instance, as Figure 6 and Figure 7 show, fibrinogen and albumin concentrations in the changing outlet velocity models reached higher values in the TEVG intima surface that occur faster compared to the constant outlet velocity models. As previously stated, at t = 0.3 s, maximum TEVG surface fibrinogen concentration is close to 7 × 10^−3^ mol/m^3^ with an average concentration of 3 × 10^−3^ mol/m^3^, for the changing outlet velocity model (Figure 6A). At the same time step, the maximum TEVG surface fibrinogen concentration is close to 3 × 10^−3^ mol/m^3^ with an average protein concentration of 1 × 10^−3^ mol/m^3^ for the constant outlet velocity model (Figure 5A).

Similarly, at t = 0.3 s, the maximum TEVG surface albumin concentration is about 0.6 mol/m^3^, with an average concentration of 0.3 mol/m^3^, for the changing outlet velocity model (Figure 7A). At t = 0.3 s, maximum TEVG surface albumin concentration is 0.3 mol/m^3^, with an average protein concentration of 0.15 mol/m^3^, for the constant outlet velocity model (Figure 5C). The differences in maximum and average protein concentrations between constant outlet velocity models compared to time and spatial dependent outlet velocity models are most likely due to the imposed velocity boundary conditions, which impact the time-scale and distribution of proteins on the TEVG surface.

In addition, the concentration profiles along the geometry are considerably less homogeneous in the changing outlet velocity model compared to the constant velocity one. The changing outlet velocity models (Figure 6 and Figure 7) show wider concentration ranges when compared to the constant velocity one (Figure 5), suggesting a strong dependence to not only initial protein concentration but also to pulsatile flow and spatial-dependent velocity variables.

From the above, our model suggests that the spatial and temporal distribution of proteins are closer to those obtained experimentally when the pulsatile flow resulting from the two-way coupled with the TEVG wall deformation is taken into account.

To our knowledge, the mechanistic understanding of protein adsorption on TEVG’s intima has been limited by the oversimplification of flow conditions i.e., by considering constant physiological average velocity and pressure conditions. However, our results suggest that f flow properties such as pulsatile flow and the TEVG’s wall resistance to circumferential and radial deformation under pulsatile pressure conditions, are also critical in defining protein adsorption.

Our model provides a robust tool to support the rational design and study of TEVGs under physiological-like conditions before in vivo evaluation. Since albumin and fibrinogen adsorption on TEVG is correlated to the thrombotic response and modulates subsequent cells-surface interaction, our model provides a novel tool for predicting the TEVGs’ biocompatibility and possible rate of success in response to variables that have been disregarded in importance previously such as the TEVG’s wall properties and the consequent surface affinity to albumin and fibrinogen.

Since our model considered the mechanical and geometrical properties of a potential scaffold for TEVG applications and a surface with adjustable affinity to proteins based on reversible and irreversible reaction constants reported previously [9,35], our model might be applied to study other biomaterials, surface modifications, and different proteins-surface interactions. Conditions altering blood flow, pressure, and vascular geometries such as hypertension, aneurysms, and atherosclerosis might also well suited for our modeling approach. Input parameters such as biomaterial mechanical properties (i.e., Young Modulus and Poisson ratio), TEVG geometry, fluid properties, pressure and velocity boundary conditions, and protein characteristics (i.e., protein size, and concentration) can be adjusted to the particular needs of an intended application.

However, the model is limited to laminar flow conditions and incompressible fluid, which might not be realistic for a number of applications. The model considers a single protein injection within the TEVG segment, which differs from in vivo conditions where proteins are continuously circulated in the bloodstream. Besides, the model fails to evaluate the effect of varying protein electrical properties on protein adsorption and also neglects the anisotropy of charge distribution on the protein surface [42,43,44,45,46]. For this reason, a deeper understanding of the interplay of electrochemical variables under the physiological-like flow conditions could be valuable to provide further insights into the rational design of novel surface coatings capable of decreasing thrombogenesis and promoting cell adhesion.

To improve the model validity, the structural component of the TEVG wall mechanical model should be refined and geometrical details such as native artery microgrooves and sudden expansions and contractions along the geometry should be incorporated [28]. Protein properties such as shape, and surface-protein-fluid conditions such as the charge and active site distribution along the graft need to be considered comprehensively to improve model accuracy. In summary, the presented multiphysics model suggested differences in protein adsorption under constant velocity compared to the time and spatial dependent velocity condition at the outlet that results in a faster fibrinogen coating compared to albumin under human artery hemodynamic settings. This result corroborates that protein adsorption is highly sensitive to flow conditions [30] mainly regulated by the mechanical and protein-surface properties of the designed TEVG and thus, indicates that the proposed model could serve as a useful tool of in silico validation of novel TEVG performance prior in vitro and in vivo studies.

## 4. Materials and Methods

### 4.1. Two-Way Fluid-Structure Interaction Model

A two-way Fluid-Structure Interaction model (two-way FSI) coupled to a Multiphysics model for species transport was used for the in silico modeling of protein adsorption on TEVGs involving mechanical, hemodynamic, and biochemical parameters. Together with an experimental setup of pulsatile flow according to our previous approach of a fluidic device for TEVGs testing [28]. Then, wall deformation by fluid dynamics were included to avoids overestimating flow rates [47] and allows exploring dynamic operation conditions closely to the physiological phenomena.

### 4.2. Structural Model

**Figure 8:**
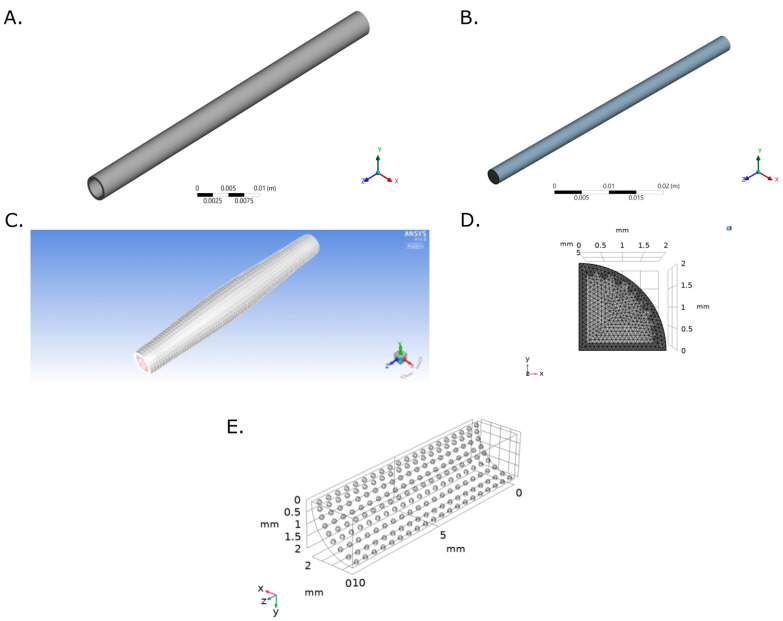
Computational domain and selected mesh for (**A**) Transient Structural and (**B**) Flow Model of a TEVG based on a Decellularized Porcine Carotid Artery. (**C**) Dynamic mesh for the last time step with a Diffusion Parameter of 1. (**D**) Multiphysics model computational domain and Selected mesh for the computational domain. (**E**) The mesh consisted of a total of 901,411 triangular and tetrahedral elements.

Transient structural (TS) model was simulated in ANSYS TS from ANSYS Workbench 19.0. A new material was created in the Engineering Data module with a Young’s Modulus of 1.34 MPa obtained from uniaxial tensile strength tests of Decellularized Carotid Porcine Artery-based TEVG [48] following the ISO 7198:2016 standard. A Poisson ratio of 0.49 was used, as reported for native blood vessels [49]. The computational domain and the selected mesh for a 4.00 mm internal diameter, 0.90 mm of thickness and 60.00 mm length graft are shown in Figure 8A.

**Figure A1:**
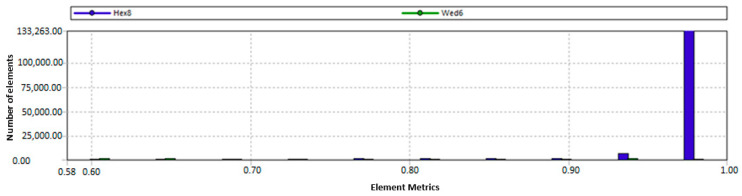
Orthogonality as mesh quality indicator for fluid TEVG domain.

**Figure A2:**
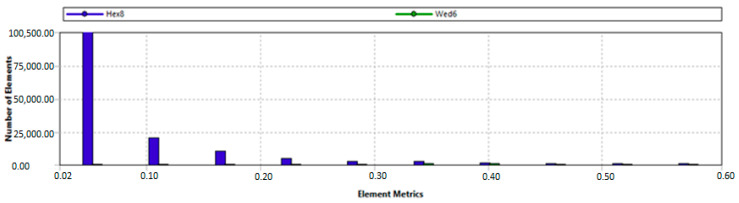
Skewness as mesh quality indicator for fluid TEVG domain.

**Figure A3:**
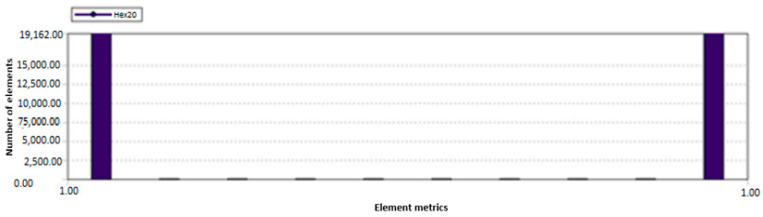
Orthogonality as mesh quality indicator for TEVG wall domain.

**Figure A4:**
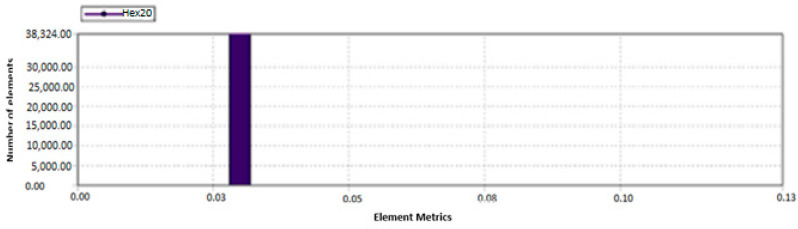
Skewness as mesh quality indicator for TEVG wall domain.

Face meshing was conducted over the inlet face with a body sizing of 2.125 × 10^4^ m. Mesh convergence analysis was obtained from inlet mass flow rate for five mesh configurations obtained by modifying their element size. A mesh consisting of 38,324 elements was then selected and the mesh quality metrics are shown in Appendix A (Figure A1, Figure A2, Figure A3 and Figure A4). Fixed support constrains were assigned to the inlet and outlet faces and a Fluid-Solid Interface was assigned to the TEVG internal face. On Analysis Settings, Auto Time Stepping function was turned off. A Step End Time of 1.50 s and a Time Step of 0.003 s were used. Large Deflection and Files Retention after Full Solve functions were allowed and Total Deformation, Equivalent Stress and Equivalent Elastic Strain were recorded.

### 4.3. Flow Model

**Figure A5:**
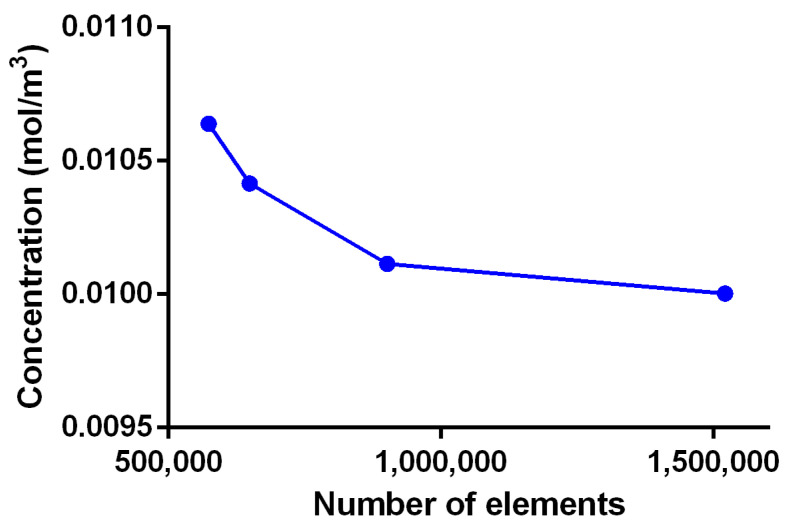
Mesh convergence analysis for adsorption model in COMSOL Multiphysics.

**Figure A6:**
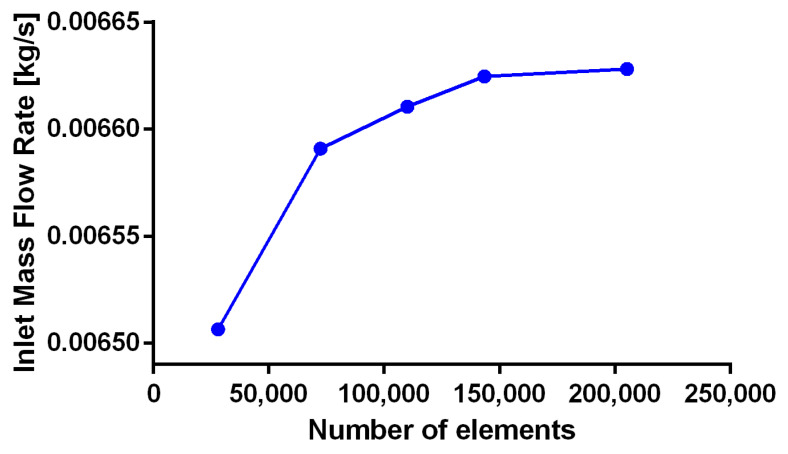
Mesh convergence analysis for fluid TEVG domain at an outlet probe point for t = 0.36 s.

The fluid computational domain consisted of a 4.00 mm diameter and 60.00 mm length cylinder (Figure 8). Inflation mesh function was used at the inlet face while Face Meshing was applied to the external cylinder (TEVG Wall) with a Body Sizing with an element size of 3 × 10^−4^ (Figure 8B). A mesh with a total of 143,313 elements was selected after mesh convergence analysis (Appendix B Figure A5 and Figure A6).

Laminar flow was selected according to the calculated Reynolds number of 106.09. Macroscopic experimental pressure drop of 8.48 Pa was calculated according to the Hagen-Poiseuille equation considering the distance between pressure transducers (TEVG length) and the TEVG radius. A pressure gradient of 3.12 Pa was obtained for a TEVG length of 60.00 mm and the Navier-Stokes equations were solved by the numerical method (Equations (1)–(3)).

(1) $$\nabla \cdot \vec{V}=0\;\left(Continuity\;Equation\right)$$

(2) $$\rho \left(\frac{\partial \vec{V}}{\partial t}+\left(\vec{V}\cdot \nabla \right)\vec{V}\right)=-\nabla p+\mu \nabla^{2}\vec{V}\;\left(Conservation\;of\;Momentum\right)$$

(3) $$\begin{array}{c} \frac{1}{r}\frac{\partial \left(ru_{r}\right)}{\partial r}+\frac{\partial u_{z}}{\partial z}=0\\ \rho \left(\frac{\partial u_{r}}{\partial t}+v_{r}\frac{\partial u_{r}}{\partial r}+v_{z}\frac{\partial u_{r}}{\partial z}\right)=-\frac{\partial P}{\partial z}+\mu \left(\frac{1}{r}\frac{\partial }{\partial r}\left(r\frac{\partial u_{r}}{\partial r}\right)-\frac{u_{r}}{r^{2}}+\frac{\partial^{2}u_{r}}{\partial z^{2}}\right)\\ \rho \left(\frac{\partial u_{z}}{\partial t}+u_{r}\frac{\partial u_{z}}{\partial r}+u_{z}\frac{\partial u_{z}}{\partial z}\right)=-\frac{\partial P}{\partial z}+\mu \left(\frac{1}{r}\frac{\partial }{\partial r}\left(r\frac{\partial u_{z}}{\partial r}\right)+\frac{\partial^{2}u_{z}}{\partial z^{2}}\right) \end{array}$$

Experimental inlet and outlet experimental pulsatile pressure data was processed in MATLAB R2018a using *fft*() function to reconstruct the original signal into Discrete Fourier Transform functions (DFT, Equation (4)) as previously reported [28], imported to ANSYS Fluent 19.0 as User Defined Functions (UDF) and, assigned as Inlet and Outlet faces boundary conditions. The resulting functions for pressure were written on a C script (Supplementary Material Table S1).

(4) $$P_{in,\;out}\left(t\right)=A_{o\;in,\;out}+\left(A_{n\;in,\;out}*cos\left(\omega *t*n\right)\right)+\left(B_{n\;in,\;out}*sin\left(\omega *t*n\right)\right)$$

where ω= 0.8184 rad/s, *n* = 125 harmonics ($A_{n}$ and $B_{n}$) were included for inlet and outlet pressure functions reconstruction. $A_{o}$ corresponded to 11,819 Pa for inlet pressure and 12,784 Pa for outlet pressure. The flow model was set as transient and pressure-based. A Dynamic mesh was enabled by implementing the Smoothing- Diffusion—Boundary distance Method with Diffusion [50] with a diffusion parameter of 1.0 since it provided better results for mass flow rate. Two dynamic mesh were created: a deforming zone for the fluid domain and a system coupling zone for fluid-TEVG contact wall (Figure 8C).

The model was solved under a SIMPLE scheme for Pressure-Velocity coupling, a Second Order Upwind for momentum and a Least Squares Cell Based method and PRESTO method for Spatial Discretization and for pressure, respectively. The transient formulation was solved under a First Order Implicit method and a report definition were created for mass flow rate at the inlet and outlet. Residuals Absolute Criteria were set at 1 × 10^−6^ for continuity for x, y and z-equations collecting data each two-time steps after a standard initialization with time step of 0.003 s for a total simulation time of 1.5 s.

### 4.4. Fluid and Transient Structural Models Coupling

System coupling was added to the ANSYS Workbench project. Setup from Transient Structural (TS) and Fluid Flow (Fluent) were linked to the System Coupling setup with End Time of 1.5 s and a Step Size of 0.003 s. The model was run transiently with a program-controlled coupling initialization and ten maximum iterations. On the Fluid Solid Interface Region in Transient Structural force was considered as input and displacement as output variables, while for the external fluid wall in the Fluid Flow model displacement was considered as input and force as output.

Because the pulsatile pressure was not directly set up in the transient structural model, the coupling between the fluid flow and the structural model was verified by the presence of pulsatile deformations over time. Two Data sets were transferred between the Fluid-Solid Interface in TS and TEVG-fluid wall interface. The first Data Transfer included a source variable of Incremental displacement on Transient Structural and a target variable of displacement on Fluid Flow, while the second Data Transfer considered force as source variable in the Fluid Flow model and Force as target variable in the TS model. The solving sequence was first assigned to the TS model followed by the Fluid Flow one. The model validation was obtained by comparison with the experimentally obtained flow rate using the first twenty-five time steps.

### 4.5. Protein Adsorption on TEVG: Biochemical Parameters

The impact of biochemical parameters on protein adsorption were evaluated by a single-component protein solution for albumin and fibrinogen adsorption over the TEVG surface in COMSOL Multiphysics 5.5. The surface reaction dynamics for reversible and irreversible protein adsorption introduced by Latour et al. [9] were adapted (Equation (5)) in the presence of flow (Figure 2A) using a continuous stirred tank reactor (CSTR) and, introduced as differential equations to capture products and reactants concentration profiles over time (Equations (6)–(9)). Briefly, a protein *P* interacts with the surface sites for adsorption *S* in a constant rate *k_f_* to become a reversible complex *P∙S* by hydrolysis. Then, the complex changes their conformation into a bulk irreversible compound $\bar{P}\cdot \bar{S}$ a rate constant *k_i_* by a second hydrolysis or return to their initial state at a constant rate *k_i_* by condensation.

The parameters *m*, *n*, *r* and *v* represent the water molecules available for each reaction and were used as control for stoichiometric balance. Accordingly, before the reaction, a total of m+r+n+v  water molecules are released as bulk water [51].

(5) $$\begin{array}{l} P\cdot \left(m+n\right)H_{2}O+S\cdot \left(r+v\right)H_{2}O\;\begin{array}{c} \overset{k_{f}}{\to }\\ \overset{k_{r}}{\leftarrow } \end{array}P\cdot S\cdot nH_{2}O\\ +\left(m+r\right)H_{2}O\overset{k_{i}}{\cdot }\bar{P}\cdot \bar{S}+\left(n+v\right)H_{2}O \end{array}$$

(6) $$\frac{d\left[P\cdot S\right]}{dt}=k_{f}\left[P\right]\left[S\right]-k_{r}\left[P\cdot S\right]-k_{i}\left[P\cdot S\right]$$

(7) $$\frac{d\left[\bar{P}\cdot \bar{S}\right]}{dt}=k_{i}\left[P\cdot S\right]$$

(8) $$\frac{d\left[S\right]}{dt}=-k_{f}\left[P\right]\left[S\right]+k_{r}\left[S\right]$$

(9) $$\frac{d\left[P\right]}{dt}=-k_{f}\left[P\right]\left[S\right]+k_{r}\left[P\right]$$

**Figure A7:**
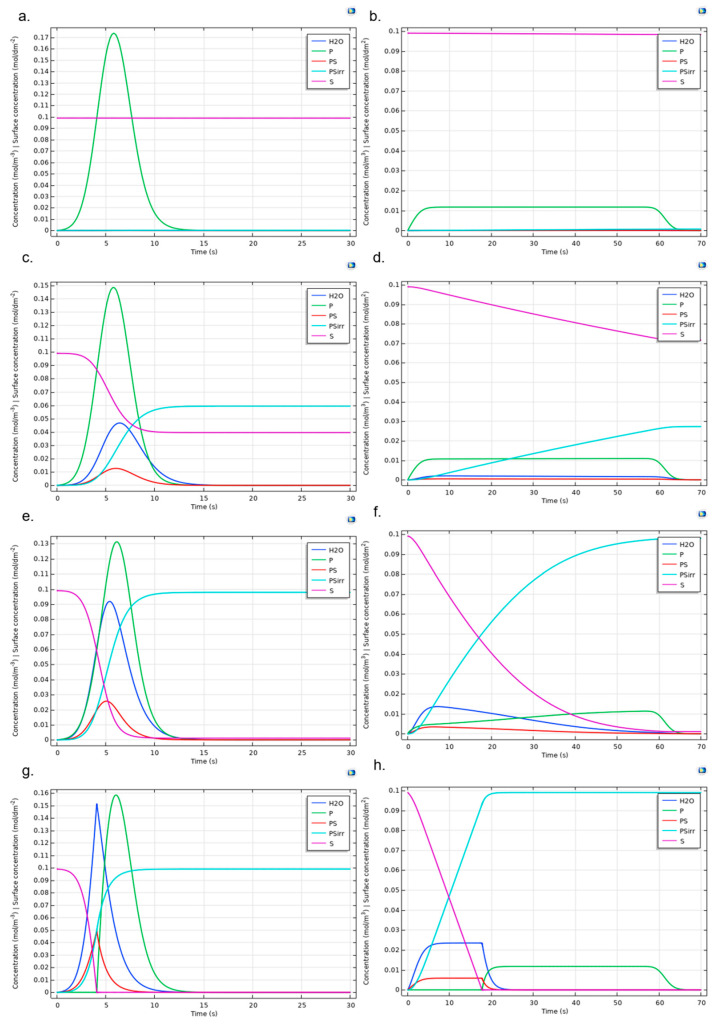
Protein adsorption reaction dynamic for Albumin (left column) and Fibrinogen (right column). Figures show the cases *k_r_* = 1, *k_i_* = 1 and (**a**,**b**) *k_f_* = 0.001, (**c,d**) *k_f_* = 0.5, (**e**,**f**) *k_f_* = 9, and (**g**,**h**) *k_f_* = 1 × 10^6^. Surface concentrations are scaled (1 × 10^4^ times) to allow comparison with bulk species.

**Figure A8:**
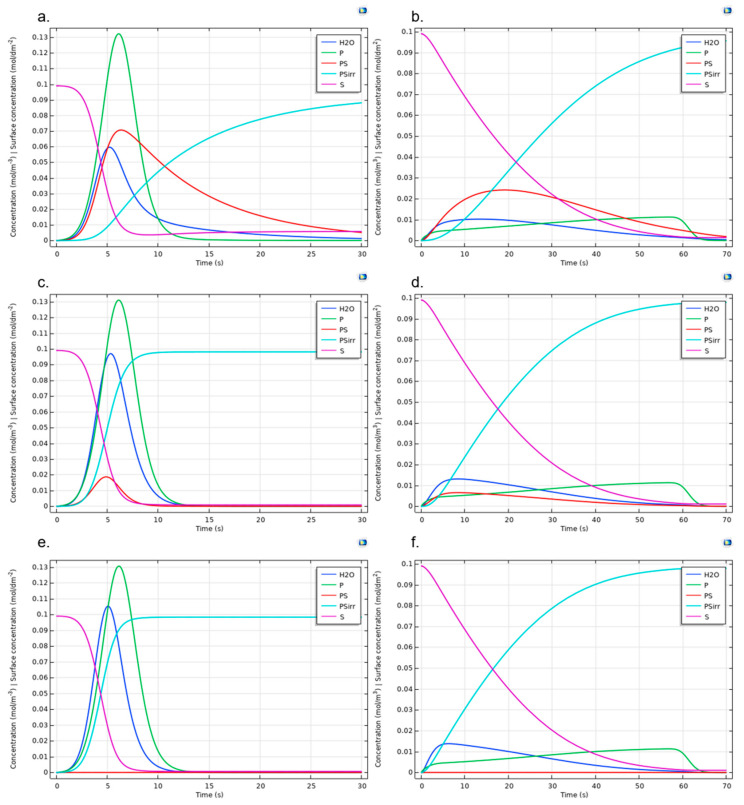
Protein adsorption reaction dynamic for Albumin (**left column**) and Fibrinogen (**right column**). Figures show the cases *k_r_* = 1, *k_f_* = 9 and (**a**,**b**) *k_i_* = 0.1, (**c**,**d**) *k_i_* = 1.5, and (**e**,**f**) *k_i_* = 1 × 10^4^. Surface concentrations are scaled (1 × 10^4^ times) to allow comparison with bulk species.

The reactions dynamics were studied using the Reaction Engineering physic for both proteins using the parameters presented in Table 1 after a sensitivity analysis of the kinetic variables (Appendix C Figure A7 and Figure A8). Briefly, $k_{r},\;k_{f},\;k_{i}\;$ were selected based on the physicochemical properties of both proteins and the surface charge of the TEVG (assumed to be composed mainly by collagen) that favors or inhibits external interactions between the protein motifs with the hydrophobic surface [9].

**Figure 9:**
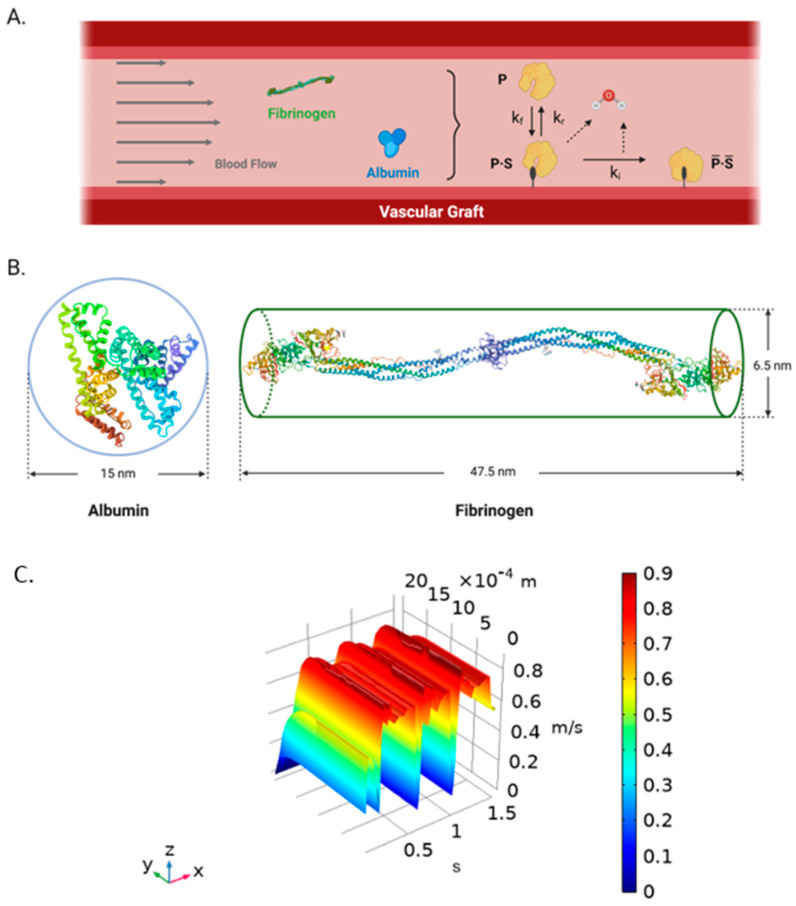
(**A**) Schematic representation of the biochemical surface reactions dynamics for single-component protein adsorption on TEVGs [9]. A protein (P) interacts with the surface sites for adsorption (S) in a constant rate *k_f_* to become a reversible complex (P∙S) by hydrolysis. Then, the complex changes their conformation into a bulk irreversible compound $\bar{P}\cdot \bar{S}$ a rate constant ki by a second hydrolysis or return to their initial state to the blood flow at a constant rate ki. (**B**) Albumin (**left**) (Modified from PBD, Sugio, et al., 1999 [52]) and fibrinogen (**right**) (Modified from PBD, Kollman et al., 2009 [26]) simplification as spheroid and cylindrical prism, respectively. (Created with BioRender.com, accessed on 22 September 2021) (**C**) Time and spatial dependent velocity surface function obtained from the two-way FSI model in ANSYS and imported into COMSOL Multiphysics 5.5^®^ using an interpolation function. *X*-axis corresponds to time (s), *Y*-axis corresponds to the radial direction (radius) (m), and the *Z*-axis is the velocity dependent variable (m/s).

Albumin was assumed to have spherical shape with diameter of 8.50× 10^−3^ µm while fibrinogen was assumed to be cylindrical with a cross-sectional area of 6.50 × 10^−4^ µm^2^ and length of 4.75 × 10^−3^ µm (Figure 9B) to enable species tracking in the reaction aiming to simplify the calculations of volume and density. Temperature was stablished according to the standard human physiological value (310 K), the initial concentrations of reaction products were set at 0 mol/m^3^ and one site of occupancy was assigned to each surface species.

A 3D analysis of transport of diluted species over a surface reaction with a laminar flow by coupling the Chemistry, the Transport of Diluted Species, the Surface Reaction and the Laminar Flow physics were performed (Appendix D). The computational domain was set as the TEVG used in the two-way FSI model assuming symmetry in a longitudinal quarter with reaction sites represented as spheres of 0.1 mm of diameter at the internal surface (Figure 8D).

The reaction sites were meshed using triangular elements with a minimum and maximum element size of 1.00 × 10^−3^ mm and 1.00 × 10^−3^ mm, while the geometry domain was meshed with tetrahedral elements with a minimum and maximum element size of 1.00 × 10^−3^ mm and 1.00 × 10^−3^ mm (Figure 8E). Mesh convergence analysis was performed for five mesh configurations with several elements between 573,562 and 1,520,933 using the concentration of fibrinogen as reference at (0.75, 0.75, 5) mm and t = 0.0195 s. The convergence criteria corresponded to an error between mesh configurations below to 0.01% which indicate an optimal mesh of 901,411 elements.

### 4.6. Two-Way FSI and Protein Adsorption Models Coupling

The velocity profiles from the two-way FSI were obtained at outlet line with 22 spatial points for 0 to 1.5 s with a time step of 0.009 s as function of the radial position (Figure 9C). Then, the time-spatial dependent velocity data was imported into COMSOL Multiphysics 5.5 using the Nearest Neighbor Interpolation function with two arguments from x = 0 to x = 2 mm, due to the symmetrical assumption and ran for 1.5 s with a 0.009 s time step. Time and position variables were declared, and the interpolated velocity function was assigned to the outlet face as the boundary condition.

## 5. Conclusions

Two-way FSI models provided fully developed velocity profiles and a physiological baseline of wall shear stress distributions compared to previous Rigid Wall Models demonstrating the relevance of considering structural deformation to obtain accurate results. This protein adsorption model has a high sensitivity to biochemical and flow parameters. It was possible to validate the proposed computational methods to further gain insights into the governing phenomena of TEVG under physiological conditions. Hydrophobicity and surface charge are the main parameters to modulate protein adsorption according to its size, concentration and aminoacidic-based structure. Therefore, our computational models provide a robust platform to multiparametrically study the performance of novel surface modifications on TEVGs, as a useful tool to control protein adsorption.

Finally, the presented computational methodology can be potentially extended to the analysis of the effect of hemodynamic variables over the adsorption of plasma proteins related to the performance of TEVGs to improve endothelialization in protein-coated surfaces. Then, material and surface biochemical modifications screening by our multiphysics in-silico could be included in the rational design of TEVGs after measuring their hydrophobicity and mechanical properties reducing the number of in-vitro experiments and animal specimens needed to test safety and functionality prior clinical trials.

## Figures and Tables

**Table 1:** Fibrinogen and Albumin adsorption parameters on the COMSOL Multiphysics model.

Parameter	Value	Units	Description
R_Area_	$2.00\times 10^{4}$	m^2^	Surface reaction area
Inlet_Flow	$3.33\times 10^{-7}$	m^3^/s	Inlet flow rate
*k_f_*	9	m^3^/(s mol)	Forward rate constant
*k_r_*	1	m^3^/(s mol)	Reverse rate constant
*k_i_*	$1\times 10^{-4}$	L/s	Forward rate constant for irreversible reaction
CF_max_inlet_	0.0118	mol/m^3^	Maximum concentration fibrinogen
CA_max_inlet_	0.753	mol/m^2^	Maximum concentration albumin
CS_0surf_	9.90 × 10^−6^	mol/m^2^	Initial surface concentration *S*
CH_2_O	55,600	mol/m^3^	Concentration of solvent (water)
G_0_	$1\times 10^{-5}$	mol/m^2^	Initial site density of S
MA	66.5	kg/mol	Molar mass Albumin
MF	340	kg/mol	Molar mass Fibrinogen
MS	0.018	kg/mol	Molar mass water
MH_2_O	0.018	kg/mol	Molar mass water
ρ H_2_O	1000	kg/m^3^	Density water
ρ A	70.69	kg/m^3^	Density Albumin
ρ F	35.82	kg/m^3^	Density Fibrinogen
μ H_2_O	$1\times 10^{-3}$	Pa∗s	Dynamic viscosity water
Velocity	V(x,t)	m/s	From ANSYS velocity in the TEVG
MPS	66.5 (340)	kg/mol	Molar mass P·S
MPS_irr_	66.5 (340)	kg/mol	$\mathrm{Molar}\;\mathrm{mass}\;\bar{P}\cdot \bar{S}$
Time step	0.009	s	Discretization time step for the simulations

## Data Availability

The data presented in this study are available on request from the corresponding author.

## Supplementary Materials

The following supporting information can be downloaded at: https://www.mdpi.com/article/10.3390/ijms23031130/s1, available on: https://drive.google.com/drive/folders/1i0R3sRptIcw2KckQRCWrCHm0wP1WNrGO?usp=sharing (accessed on 15 December 2021).

## Appendix A

- **Mesh quality ANSYS models**

## Appendix B

- **Mesh convergence analysis**

## Appendix C

- **COMSOL sensibility analysis and additional time steps**

The reactions dynamics were first studied in 0D using the Reaction Engineering physic for both proteins using the parameters presented in Table 1. Fifty inlet Gaussian pulses with a standard deviation of 1.5 were used to introduce the proteins and the solvent to the reaction as a simplification of a single heartbeat. Albumin was assumed to have spherical shape with diameter of 8.50 × 10^−3^ µm while fibrinogen was assumed to be cylindrical with a cross-sectional diameter of 6.50 × 10^−4^ µm and length of 4.75 × 10^−3^ µm.

For the sensitivity study of the kinetic variables in the CSTR, temperature was stablished according to the standard human physiological value (310 K). The initial concentrations of reaction products were set at 0 mol/m^3^. One site of occupancy was assigned to each surface species. The reversible and irreversible reactions kinetics were modeled according to Equations (A1)–(A4) with a forward rate constant *k_f_* between 0.001 to 100,000 s^−1^, and a fixed reverse rate constant *k_r_* of one.

(A1) $$V_{r}\frac{dc_{j}}{dt}=\underset{m}{\sum }v_{f,m}c_{f,mj}-vc_{j}+V_{r}R_{j}+A_{r}R_{\mathrm{ads},i}$$

(A2) $$\frac{dV_{r}}{dt}=0$$

(A3) $$v=\underset{m}{\sum }v_{\;f,m}+v_{\;p}$$

(A4) $$\frac{dc_{j}}{dt}=R_{j}=v_{j}r$$

The range for the forward rate constant was obtained from the experimental values reported by Latour for saturation percentage and initial free energy of adsorption $\left(\Delta G_{ads}^{0}\right)$ and its corresponding $KC_{e}$ value for fibrinogen [9]. The equilibrium equation for $\Delta G_{ads}^{0}$ is presented in Equation (A5) as function of the equilibrium constant (K), the temperature (*T*) and the ideal gas constant (*R*). The forward rate constant for the irreversible reaction k*_irr_* was evaluated in a range of 1 × 10^−3^ to 1 × 10^6^ by establishing *k_f_* as 9 and *k_r_* as 1, in accordance with the Equation (A6) under a $\Delta G_{ads}^{0}$ of −9.20 kcal/mol and a saturation percentage of 90% [9]. The range of values for *k_f_* and *k_i_* were reported by Latour according to the $\Delta G_{ads}^{0}$, which depends on the number of hydrogen bonds, or the possible hydrophobic interactions and the energy required for their formation, i.e., −3.5 kcal/mol (which is required for the formation of a hydrogen bond) [9].

(A5) $$\Delta G_{ads}^{0}=-RT\;ln\left(K\right)$$

(A6) $$K=\frac{K_{f}}{K_{r}}$$

## Appendix D

- **Multiphysic model setup details**

*1. Chemistry:*

Surface reaction equations were obtained from the Reaction Engineering component (Equations (A7) to (A9)). Parameters such as molar mass and density of the protein, surface species, solvent and protein-surface species are presented in the Table 1.

*2. Transport of Diluted Species:*

The transport mechanism corresponded to mass transport through the longitudinal axis of the TEVG from the inlet through the outlet cross-sectional areas. The governing equation was the conservation of species, which is presented in Equation (A7). Equation (A8) is the surface reaction and Equation (A9) is the boundary condition that corresponded to a cero flux across the wall of the geometry, across the symmetric walls and inside the reaction sites (spheres).

(A7) $$\begin{array}{l} \frac{\partial c_{i}}{\partial t}+\nabla \cdot J_{j}+u\cdot \nabla c_{j}=R_{j}\\ J_{j}=-D_{j}\nabla c_{j} \end{array}$$

(A8) $$-n\cdot J_{i}=J_{0,j}$$

(A9) $$-n\cdot J_{i}=0$$

An initial value of 1 × 10^−7^ mol/m^3^ was set for the bulk protein concentration and the released water molecules, with the purpose of providing more stability to the simulation. Inflow was set on the transversal faces of the vessel while outflow was set on the opposite face. The maximum concentration of the protein was set on the inlet face of the vessel as an initial condition. The initial concentration of released water molecules was established as zero. A Danckwerts flux boundary condition type was imposed for the inflow. For the outlet face the flux is described by Equation (A10).

(A10) $$n\cdot D_{j}\nabla c_{j}=0$$

*3. Surface Reactions*

The surface reactions were assigned to the reaction sites or spheres of 0.1 mm on the internal surface of the vessel. The governing equations for the surface reactions are shown below (Equations (A11)–(A14)).

(A11) $$\nabla_{t}\cdot \left(-D_{j}\nabla_{t}c_{s,j}\right)=R_{s,j}$$

(A12) $$N_{s,j}=-D_{j}\nabla_{t}c_{s,j}$$

(A13) $$\theta_{j}=\frac{c_{s,j}\sigma_{j}}{\Gamma_{s}}$$

(A14) $$\frac{\partial c_{b,j}}{\partial t}=R_{b,j}$$

where Γ_s_ correspond to the site density and its value is presented as G0 in the parameters in Table 1, σ_cs_ is the site occupancy number for the surface reactive sites *S* and for the reversible and irreversible protein-surface complexes. Also, the parameter was assigned as one for the surface species. The initial values for surface concentration were established as the parameter CS0surf in the parameters Table 1 for the surface specie *S*. A small initial amount of surface concentration for the reversible and irreversible protein-surface complexes was calculated as 0.01 times the CS0surf parameter.

*4. Laminar flow*

An incompressible flow condition was considered for the simulation. The Navier-Stokes equations and the continuity equation were solved for the model. The fluid was assumed as Newtonian with the dynamic viscosity presented in the Table 1. Equations (4) and (5) were the governing relation for the laminar flow. The No slip condition was imposed on the TEVG walls (Equation (A15)). The inlet and outlet boundary conditions are explained above in the Models Coupling section.

(A15) $${\vec{u}}_{wall}=0$$

A symmetric condition was imposed on the internal walls of the quarter vessel (Equations (A16) and (A17)).

(A16) u⋅n=0

(A17) $$K_{n}-\left(K_{n}\cdot n\right)n=0,K_{n}=Kn$$

A study with a stationary first step and a subsequent temporal step was used to solve the model. The stationary step solved the equations for the Laminar flow, while the temporal step solved the Chemistry, Transport of Diluted Species and Surface Reactions ones. Multiphysics coupling between Reacting Flow and Diluted Species was allowed for both studies. The FMRES method was used to solve the model with a relative tolerance of 2 × 10^−1^ for the time dependent model.
