# Supplementary material for: Computational Characterization of Mechanical, Hemodynamic, and Surface Interaction Conditions: Role of Protein Adsorption on the Regenerative Response of TEVGs
Source: Int J Mol Sci. 2022 Jan 20;23(3):1130. doi: 10.3390/ijms23031130 (PMC8835378; doi:10.3390/ijms23031130)
Supplement: Supplementary file 1 [file ijms-23-01130-s001.zip › ijms-1543598-supplementary.pdf]

International Journal of *Molecular Sciences*

MDPI

Article

Computational Characterization of Mechanical, Hemodynamic, and Surface Interaction Conditions: Role of Protein Adsorption on the Regenerative Response of TEVGs

Alejandra Riveros [1,*], Andres J. Garcia-Brand [1], Maria A. Rodriguez-Soto [1], Nestor Sandoval [2], Carolina Muñoz-Camargo [1], Juan C. Cruz [1,*] and Juan C. Briceño [1,3,*]

[1] Department of Biomedical Engineering, Universidad de los Andes, Bogotá 11711, Colombia; aj.garcia14@uniandes.edu.co (A.J.G.-B.); ma.rodriguezs1@uniandes.edu.co (M.A.R.-S.); c.munoz2016@uniandes.edu.co (C.M.-C.)
[2] Department of Congenital Heart Disease and Cardiovascular Surgery, Fundación Cardio Infantil Instituto de Cardiología, Bogotá 111711, Colombia; nsandoval@cardioinfantil.org
[3] Department of Research, Fundación Cardio Infantil Instituto de Cardiología, Bogotá 111711, Colombia
* Correspondence: ra.riveros11@uniandes.edu.co (A.R.); jc.cruz@uniandes.edu.co (J.C.C.); jbriceno@uniandes.edu.co (J.C.B.); Tel.: +57-(1)-339-4949 (ext. 1789) (J.C.B.)

Citation: Riveros, A.; Garcia-Brand, A.J.; Rodriguez-Soto, M.A.; Sandoval, N.; Muñoz-Camargo, C.; Cruz, J.C.; Briceño, J.C. Computational Characterization of Mechanical, Hemodynamic, and Surface Interaction Conditions: Role of Protein Adsorption on the Regenerative Response of TEVGs. *Int. J. Mol. Sci.* **2022**, *23*, 1130. https://doi.org/10.3390/ijms23031130

Academic Editor: Masaru Tanaka

Received: 22 December 2021
Accepted: 15 January 2022
Published: 20 January 2022

Publisher's Note: MDPI stays neutral with regard to jurisdictional claims in published maps and institutional affiliations.

Copyright: © 2022 by the authors. Submitted for possible open access publication under the terms and conditions of the Creative Commons Attribution (CC BY) license (https://creativecommons.org/licenses/by/4.0/).

A4. Additional times for Two Way FSI and Multiphysics models are available on:

https://drive.google.com/drive/folders/1i0R3sRp-tIcw2KckQRCWrCHm0wP1WNrGO?usp=sharing

A5. *C code* of inlet and outlet pressure UDF from DFT experimental pressure function reconstruction

```
/*********************************************************
unsteady.c
UDF FOR SPECIFYING A PULSATILE PRESSURE PROFILE BOUNDARY CONDITION
**********************************************************************/
#include "udf.h"

DEFINE_PROFILE(unsteady_pressureIN,thread,position)
{
 face_t f;
 real t= CURRENT_TIME;
 begin_f_loop(f,thread)
{
      F_PROFILE(f,thread,position)=

11984+
((11.5848190353309*cos(0.8184*t*1))+(0.505073010755772*sin(0.8184*t*1)))+
((18.1803330251078*cos(0.8184*t*2))+(-10.2282506216021*sin(0.8184*t*2)))+
((8.20697838130978*cos(0.8184*t*3))+(-10.0868024426746*sin(0.8184*t*3)))+
((7.68579892580832*cos(0.8184*t*4))+(-7.0201712233807*sin(0.8184*t*4)))+
((11.3177822014377*cos(0.8184*t*5))+(-6.10447302102477*sin(0.8184*t*5)))+
((12.1172783300953*cos(0.8184*t*6))+(-4.46469139877595*sin(0.8184*t*6)))+
((14.883055602792*cos(0.8184*t*7))+(-6.76676672454061*sin(0.8184*t*7)))+
((17.3497682113768*cos(0.8184*t*8))+(-7.97306857926715*sin(0.8184*t*8)))+
((27.2582181444711*cos(0.8184*t*9))+(-18.1897267934749*sin(0.8184*t*9)))+
((-6.85711814770048*cos(0.8184*t*10))+(22.9475188779767*sin(0.8184*t*10)))+
((11.5362592331759*cos(0.8184*t*11))+(4.01419021466047*sin(0.8184*t*11)))+
((16.6851906963304*cos(0.8184*t*12))+(1.66908366785374*sin(0.8184*t*12)))+
```

((21.3609744467927*cos(0.8184*t*13))+(-0.00508783487603878*sin(0.8184*t*13)))+
((27.1794586557867*cos(0.8184*t*14))+(0.691896506099321*sin(0.8184*t*14)))+
((34.5160571959812*cos(0.8184*t*15))+(-0.139435079346405*sin(0.8184*t*15)))+
((47.4502644759167*cos(0.8184*t*16))+(-0.744678173963734*sin(0.8184*t*16)))+
((71.5101381766797*cos(0.8184*t*17))+(-0.426666568094682*sin(0.8184*t*17)))+
((133.263501354685*cos(0.8184*t*18))+(0.683034643440251*sin(0.8184*t*18)))+
((681.684336749673*cos(0.8184*t*19))+(7.37694311909448*sin(0.8184*t*19)))+
((-240.295211605516*cos(0.8184*t*20))+(-8.10943095509203*sin(0.8184*t*20)))+
((-105.999336539911*cos(0.8184*t*21))+(-5.62823464637175*sin(0.8184*t*21)))+
((-69.6219042357097*cos(0.8184*t*22))+(-4.54887952474233*sin(0.8184*t*22)))+
((-53.2921585322405*cos(0.8184*t*23))+(-4.89337288797861*sin(0.8184*t*23)))+
((-42.3273221657101*cos(0.8184*t*24))+(-5.01415760172456*sin(0.8184*t*24)))+
((-35.8304817722968*cos(0.8184*t*25))+(-5.2100696494674*sin(0.8184*t*25)))+
((-32.6245995664206*cos(0.8184*t*26))+(-6.19250060676557*sin(0.8184*t*26)))+
((-28.8128082226602*cos(0.8184*t*27))+(-6.44229185502621*sin(0.8184*t*27)))+
((-27.0881851383044*cos(0.8184*t*28))+(-8.15116535027251*sin(0.8184*t*28)))+
((-18.444713995999*cos(0.8184*t*29))+(5.71163481286179*sin(0.8184*t*29)))+
((-22.0760739734296*cos(0.8184*t*30))+(-6.92171620445279*sin(0.8184*t*30)))+
((-20.7949878442401*cos(0.8184*t*31))+(-8.76617429404904*sin(0.8184*t*31)))+
((-19.8092503068205*cos(0.8184*t*32))+(-10.7850298499079*sin(0.8184*t*32)))+
((-18.3726733180543*cos(0.8184*t*33))+(-12.2791334226365*sin(0.8184*t*33)))+
((-18.0057978171997*cos(0.8184*t*34))+(-15.9160694911892*sin(0.8184*t*34)))+
((-16.8093556984904*cos(0.8184*t*35))+(-21.7197135688254*sin(0.8184*t*35)))+
((-15.8972286859847*cos(0.8184*t*36))+(-31.2749407300406*sin(0.8184*t*36)))+
((-13.8435822242602*cos(0.8184*t*37))+(-52.4592113570626*sin(0.8184*t*37)))+
((-7.87377018845932*cos(0.8184*t*38))+(-157.903578870153*sin(0.8184*t*38)))+
((-25.7274874508789*cos(0.8184*t*39))+(165.386309148527*sin(0.8184*t*39)))+
((-18.8968996402921*cos(0.8184*t*40))+(55.5515595704263*sin(0.8184*t*40)))+
((-17.0254099845955*cos(0.8184*t*41))+(33.9517546548542*sin(0.8184*t*41)))+
((-16.5442564287118*cos(0.8184*t*42))+(24.7676087500265*sin(0.8184*t*42)))+
((-15.1192726379035*cos(0.8184*t*43))+(19.1789507965267*sin(0.8184*t*43)))+
((-15.1429147953492*cos(0.8184*t*44))+(15.7924148741265*sin(0.8184*t*44)))+
((-15.2295450856971*cos(0.8184*t*45))+(14.4756962594285*sin(0.8184*t*45)))+
((-14.4742873041325*cos(0.8184*t*46))+(12.5051487282413*sin(0.8184*t*46)))+
((-13.8344166178844*cos(0.8184*t*47))+(11.4798939533467*sin(0.8184*t*47)))+
((-4.86621098553198*cos(0.8184*t*48))+(15.4893927358387*sin(0.8184*t*48)))+
((-16.5424195696778*cos(0.8184*t*49))+(8.47829101382788*sin(0.8184*t*49)))+
((-15.5780845239342*cos(0.8184*t*50))+(8.41318348989221*sin(0.8184*t*50)))+
((-15.5697370157969*cos(0.8184*t*51))+(7.77442153048774*sin(0.8184*t*51)))+
((-15.2230298597481*cos(0.8184*t*52))+(7.65942371680304*sin(0.8184*t*52)))+
((-16.1104523734061*cos(0.8184*t*53))+(7.45953007053394*sin(0.8184*t*53)))+
((-16.8293790727125*cos(0.8184*t*54))+(7.32086632723692*sin(0.8184*t*54)))+
((-18.2598853946985*cos(0.8184*t*55))+(7.71808549163615*sin(0.8184*t*55)))+
((-20.7111886489946*cos(0.8184*t*56))+(8.50541497775705*sin(0.8184*t*56)))+
((-31.2127637473283*cos(0.8184*t*57))+(12.9745164924131*sin(0.8184*t*57)))+
((47.960843009909*cos(0.8184*t*58))+(-20.2365557105495*sin(0.8184*t*58)))+
((-0.489451772415908*cos(0.8184*t*59))+(-0.411476613822758*sin(0.8184*t*59)))+
((-5.25899073075925*cos(0.8184*t*60))+(1.15879997524127*sin(0.8184*t*60)))+
((-7.55837740268998*cos(0.8184*t*61))+(2.24423061516044*sin(0.8184*t*61)))+
((-8.22200365211061*cos(0.8184*t*62))+(2.07772108388226*sin(0.8184*t*62)))+
((-8.89290354588507*cos(0.8184*t*63))+(2.5197094732288*sin(0.8184*t*63)))+
((-8.93088091365632*cos(0.8184*t*64))+(2.45100025102585*sin(0.8184*t*64)))+
((-9.09995905470308*cos(0.8184*t*65))+(2.13740970460636*sin(0.8184*t*65)))+
((-9.0245506592848*cos(0.8184*t*66))+(1.09670228680153*sin(0.8184*t*66)))+

((-6.48881895755036*cos(0.8184*t*67))+(-3.16701658286968*sin(0.8184*t*67)))+
((-12.1640655523518*cos(0.8184*t*68))+(7.05081796168471*sin(0.8184*t*68)))+
((-10.847803082036*cos(0.8184*t*69))+(4.5227272516895*sin(0.8184*t*69)))+
((-11.1089502602836*cos(0.8184*t*70))+(4.01259601459023*sin(0.8184*t*70)))+
((-10.7080324771209*cos(0.8184*t*71))+(3.87214521332718*sin(0.8184*t*71)))+
((-10.8741144492822*cos(0.8184*t*72))+(3.75552695863899*sin(0.8184*t*72)))+
((-11.0192906995297*cos(0.8184*t*73))+(3.46587632618739*sin(0.8184*t*73)))+
((-10.4584555286795*cos(0.8184*t*74))+(3.59681956623282*sin(0.8184*t*74)))+
((-11.0177309139244*cos(0.8184*t*75))+(3.19928042496966*sin(0.8184*t*75)))+
((-11.3809991138873*cos(0.8184*t*76))+(3.33831931835751*sin(0.8184*t*76)))+
((-69.3298698194644*cos(0.8184*t*77))+(26.0720482956451*sin(0.8184*t*77)))+
((-9.62855923180421*cos(0.8184*t*78))+(2.95292481763248*sin(0.8184*t*78)))+
((-10.4560489646051*cos(0.8184*t*79))+(2.95326917669047*sin(0.8184*t*79)))+
((-10.0159268715691*cos(0.8184*t*80))+(3.04440342594428*sin(0.8184*t*80)))+
((-10.6373141814373*cos(0.8184*t*81))+(2.48923227579114*sin(0.8184*t*81)))+
((-10.6909366198952*cos(0.8184*t*82))+(2.32436713902283*sin(0.8184*t*82)))+
((-11.7511747152415*cos(0.8184*t*83))+(3.2753637551172*sin(0.8184*t*83)))+
((-11.5950112120887*cos(0.8184*t*84))+(2.78525272256668*sin(0.8184*t*84)))+
((-13.7046678365565*cos(0.8184*t*85))+(3.09066219665449*sin(0.8184*t*85)))+
((-19.0721686539981*cos(0.8184*t*86))+(1.94654997766728*sin(0.8184*t*86)))+
((8.63176878699876*cos(0.8184*t*87))+(5.79999989146196*sin(0.8184*t*87)))+
((-3.72077451119014*cos(0.8184*t*88))+(4.29035945586166*sin(0.8184*t*88)))+
((-5.95714374247349*cos(0.8184*t*89))+(5.34584507545606*sin(0.8184*t*89)))+
((-5.73694038092262*cos(0.8184*t*90))+(4.88409756202778*sin(0.8184*t*90)))+
((-6.24011898409634*cos(0.8184*t*91))+(4.76100823083077*sin(0.8184*t*91)))+
((-6.95622010391817*cos(0.8184*t*92))+(5.84506644505721*sin(0.8184*t*92)))+
((-5.79837563663023*cos(0.8184*t*93))+(6.69559644229484*sin(0.8184*t*93)))+
((-5.08158593463333*cos(0.8184*t*94))+(9.94393838070548*sin(0.8184*t*94)))+
((-0.392590027820302*cos(0.8184*t*95))+(16.4614248565374*sin(0.8184*t*95)))+
((30.4919724026039*cos(0.8184*t*96))+(72.8743643499508*sin(0.8184*t*96)))+
((-24.293052355973*cos(0.8184*t*97))+(-24.171923963087*sin(0.8184*t*97)))+
((-15.9388556061468*cos(0.8184*t*98))+(-9.71358956557654*sin(0.8184*t*98)))+
((-13.6527642262615*cos(0.8184*t*99))+(-6.29868511063399*sin(0.8184*t*99)))+
((-13.4978071181351*cos(0.8184*t*100))+(-4.57569497649366*sin(0.8184*t*100)))+
((-12.6481901526997*cos(0.8184*t*101))+(-2.77768896350216*sin(0.8184*t*101)))+
((-10.8863656551372*cos(0.8184*t*102))+(-0.817932318573717*sin(0.8184*t*102)))+
((-11.8140956905326*cos(0.8184*t*103))+(-2.1189586729358*sin(0.8184*t*103)))+
((-11.5788726930541*cos(0.8184*t*104))+(-0.187137195762071*sin(0.8184*t*104)))+
((-12.1138806277086*cos(0.8184*t*105))+(-0.784689095193963*sin(0.8184*t*105)))+
((2.87932949782014*cos(0.8184*t*106))+(-18.8997410545421*sin(0.8184*t*106)))+
((-8.37971616273325*cos(0.8184*t*107))+(-3.46007530205736*sin(0.8184*t*107)))+
((-8.44707119166567*cos(0.8184*t*108))+(-2.3817487208479*sin(0.8184*t*108)))+
((-8.69842443200775*cos(0.8184*t*109))+(-3.45922957593606*sin(0.8184*t*109)))+
((-9.2500104719343*cos(0.8184*t*110))+(-3.25197761091793*sin(0.8184*t*110)))+
((-8.01545697297323*cos(0.8184*t*111))+(-2.01994754990072*sin(0.8184*t*111)))+
((-8.56622144072111*cos(0.8184*t*112))+(-4.39246834632599*sin(0.8184*t*112)))+
((-6.85880093708771*cos(0.8184*t*113))+(-5.33779958504678*sin(0.8184*t*113)))+
((-2.96630060481155*cos(0.8184*t*114))+(-10.2893491319307*sin(0.8184*t*114)))+
((10.3567018060242*cos(0.8184*t*115))+(-29.498577850068*sin(0.8184*t*115)))+
((-32.976437126392*cos(0.8184*t*116))+(33.3242156976643*sin(0.8184*t*116)))+
((-18.5467979039862*cos(0.8184*t*117))+(11.4651747305617*sin(0.8184*t*117)))+
((-17.079973696753*cos(0.8184*t*118))+(7.76468178863889*sin(0.8184*t*118)))+
((-14.3478994509224*cos(0.8184*t*119))+(6.61098599091554*sin(0.8184*t*119)))+
((-13.4643369297937*cos(0.8184*t*120))+(4.87551447645859*sin(0.8184*t*120)))+

```
((-10.8689344362295*cos(0.8184*t*121))+(1.53019627766487*sin(0.8184*t*121)))+
((-14.454630851817*cos(0.8184*t*122))+(3.69913763584565*sin(0.8184*t*122)))+
((-12.8129424490451*cos(0.8184*t*123))+(3.35680940119923*sin(0.8184*t*123)))+
((-15.4340115089367*cos(0.8184*t*124))+(2.52641393700493*sin(0.8184*t*124)))+
((-21.8126549971515*cos(0.8184*t*125))+(7.71530274079081*sin(0.8184*t*125)));

 }
  end_f_loop(f,thread)
}

DEFINE_PROFILE(unsteady_pressureOut,thread,position)
{
  face_t f;
  real t= CURRENT_TIME;
  begin_f_loop(f,thread)
 {
        F_PROFILE(f,thread,position)=
11819+
((13.8408664964477*cos(0.8184*t*1))+(0.920925213547614*sin(0.8184*t*1)))+
((20.4336742854027*cos(0.8184*t*2))+(-10.2912778096531*sin(0.8184*t*2)))+
((10.162015278179*cos(0.8184*t*3))+(-9.85470101294347*sin(0.8184*t*3)))+
((10.7345145376045*cos(0.8184*t*4))+(-6.31032682518727*sin(0.8184*t*4)))+
((13.1836082565789*cos(0.8184*t*5))+(-5.60114854124748*sin(0.8184*t*5)))+
((13.9018170274191*cos(0.8184*t*6))+(-4.91479942262448*sin(0.8184*t*6)))+
((16.9119776125309*cos(0.8184*t*7))+(-6.52579961620085*sin(0.8184*t*7)))+
((19.8459382539453*cos(0.8184*t*8))+(-8.42291344784412*sin(0.8184*t*8)))+
((29.9352614287331*cos(0.8184*t*9))+(-18.5075434154291*sin(0.8184*t*9)))+
((-4.80392153385418*cos(0.8184*t*10))+(24.4255507156381*sin(0.8184*t*10)))+
((14.331821263733*cos(0.8184*t*11))+(5.17934665167207*sin(0.8184*t*11)))+
((19.3719364024258*cos(0.8184*t*12))+(2.15935167351637*sin(0.8184*t*12)))+
((24.3590595636397*cos(0.8184*t*13))+(1.52094066720457*sin(0.8184*t*13)))+
((30.6649551869431*cos(0.8184*t*14))+(1.45105162969968*sin(0.8184*t*14)))+
((39.450383473136*cos(0.8184*t*15))+(0.896453751325108*sin(0.8184*t*15)))+
((52.7524274630163*cos(0.8184*t*16))+(2.18867606761909*sin(0.8184*t*16)))+
((79.1904093020199*cos(0.8184*t*17))+(4.22679133909049*sin(0.8184*t*17)))+
((145.645602863871*cos(0.8184*t*18))+(10.0260330808088*sin(0.8184*t*18)))+
((737.823016435719*cos(0.8184*t*19))+(61.0292973975438*sin(0.8184*t*19)))+
((-258.20433468771*cos(0.8184*t*20))+(-28.6405134309485*sin(0.8184*t*20)))+
((-112.681042883453*cos(0.8184*t*21))+(-15.823467358546*sin(0.8184*t*21)))+
((-73.692457081137*cos(0.8184*t*22))+(-11.6272475649021*sin(0.8184*t*22)))+
((-54.9135385844324*cos(0.8184*t*23))+(-10.9719379359014*sin(0.8184*t*23)))+
((-44.2977585646164*cos(0.8184*t*24))+(-10.5442806244501*sin(0.8184*t*24)))+
((-37.6595688451036*cos(0.8184*t*25))+(-10.546178793995*sin(0.8184*t*25)))+
((-33.1780806362461*cos(0.8184*t*26))+(-11.4129941066935*sin(0.8184*t*26)))+
((-28.997399412734*cos(0.8184*t*27))+(-12.1263924343736*sin(0.8184*t*27)))+
((-26.8832973205162*cos(0.8184*t*28))+(-13.695612164249*sin(0.8184*t*28)))+
((-17.6187478222278*cos(0.8184*t*29))+(3.76051296210096*sin(0.8184*t*29)))+
((-20.6427025829879*cos(0.8184*t*30))+(-12.1625129458517*sin(0.8184*t*30)))+
((-19.4624619230373*cos(0.8184*t*31))+(-14.8151500612787*sin(0.8184*t*31)))+
((-17.4477613020031*cos(0.8184*t*32))+(-16.8652172269556*sin(0.8184*t*32)))+
((-15.3947242293289*cos(0.8184*t*33))+(-20.2262067660826*sin(0.8184*t*33)))+
((-13.8192290452831*cos(0.8184*t*34))+(-25.2977824361574*sin(0.8184*t*34)))+
((-10.7445208922532*cos(0.8184*t*35))+(-32.2219998161493*sin(0.8184*t*35)))+
((-6.42399051087545*cos(0.8184*t*36))+(-46.1824482689753*sin(0.8184*t*36)))+
```

((2.35753030392489*cos(0.8184*t*37))+(-75.9873618744117*sin(0.8184*t*37)))+
((44.1451687062335*cos(0.8184*t*38))+(-224.953289308472*sin(0.8184*t*38)))+
((-83.6848987075868*cos(0.8184*t*39))+(232.432096975134*sin(0.8184*t*39)))+
((-39.2684312422492*cos(0.8184*t*40))+(76.3366054806798*sin(0.8184*t*40)))+
((-29.9287857801956*cos(0.8184*t*41))+(46.138656867521*sin(0.8184*t*41)))+
((-26.1512540839123*cos(0.8184*t*42))+(32.5026352320713*sin(0.8184*t*42)))+
((-23.6845098787149*cos(0.8184*t*43))+(25.1166267372253*sin(0.8184*t*43)))+
((-23.0192954181119*cos(0.8184*t*44))+(20.5669216945061*sin(0.8184*t*44)))+
((-21.8860099226846*cos(0.8184*t*45))+(18.2094516571674*sin(0.8184*t*45)))+
((-20.6961490247336*cos(0.8184*t*46))+(15.0768489449351*sin(0.8184*t*46)))+
((-19.3029656314952*cos(0.8184*t*47))+(14.6780565256455*sin(0.8184*t*47)))+
((-7.74216778172151*cos(0.8184*t*48))+(24.0933250411111*sin(0.8184*t*48)))+
((-22.8184607717223*cos(0.8184*t*49))+(8.05665273611324*sin(0.8184*t*49)))+
((-22.4225890482641*cos(0.8184*t*50))+(7.82891870688811*sin(0.8184*t*50)))+
((-21.7210584768587*cos(0.8184*t*51))+(7.07319553617947*sin(0.8184*t*51)))+
((-22.317917309361*cos(0.8184*t*52))+(5.54488917417147*sin(0.8184*t*52)))+
((-24.0849587624171*cos(0.8184*t*53))+(4.64994733214328*sin(0.8184*t*53)))+
((-25.7973357923236*cos(0.8184*t*54))+(3.90755155436795*sin(0.8184*t*54)))+
((-30.6648502688259*cos(0.8184*t*55))+(1.6281556756253*sin(0.8184*t*55)))+
((-38.5481405173152*cos(0.8184*t*56))+(-1.25504852211077*sin(0.8184*t*56)))+
((-70.0834037126999*cos(0.8184*t*57))+(-11.962912046725*sin(0.8184*t*57)))+
((168.046329477883*cos(0.8184*t*58))+(68.2069244242351*sin(0.8184*t*58)))+
((21.7277890862826*cos(0.8184*t*59))+(17.1154575680275*sin(0.8184*t*59)))+
((7.5272152938262*cos(0.8184*t*60))+(11.5641723052948*sin(0.8184*t*60)))+
((0.631279681268111*cos(0.8184*t*61))+(9.29875298059939*sin(0.8184*t*61)))+
((-1.97594702734505*cos(0.8184*t*62))+(8.30805018491591*sin(0.8184*t*62)))+
((-4.07161590218085*cos(0.8184*t*63))+(8.37293155539119*sin(0.8184*t*63)))+
((-2.87068168583662*cos(0.8184*t*64))+(7.58503628093781*sin(0.8184*t*64)))+
((-3.66065429897907*cos(0.8184*t*65))+(6.63954712986074*sin(0.8184*t*65)))+
((-0.424467427498473*cos(0.8184*t*66))+(6.77307446359535*sin(0.8184*t*66)))+
((14.9506123662015*cos(0.8184*t*67))+(9.16591199663054*sin(0.8184*t*67)))+
((-22.9620630289905*cos(0.8184*t*68))+(3.01783623192148*sin(0.8184*t*68)))+
((-14.6869227315181*cos(0.8184*t*69))+(4.02951619080974*sin(0.8184*t*69)))+
((-12.132305466855*cos(0.8184*t*70))+(3.4254022046543*sin(0.8184*t*70)))+
((-12.1840821157912*cos(0.8184*t*71))+(2.94444054897564*sin(0.8184*t*71)))+
((-11.893460879336*cos(0.8184*t*72))+(3.93902944964142*sin(0.8184*t*72)))+
((-10.7659734343927*cos(0.8184*t*73))+(3.74419548888709*sin(0.8184*t*73)))+
((-11.5380784162063*cos(0.8184*t*74))+(3.90375595732224*sin(0.8184*t*74)))+
((-10.1611651626373*cos(0.8184*t*75))+(2.72351443665279*sin(0.8184*t*75)))+
((-9.78247853744317*cos(0.8184*t*76))+(1.66396087011278*sin(0.8184*t*76)))+
((40.7888844931922*cos(0.8184*t*77))+(-134.78653855811*sin(0.8184*t*77)))+
((-12.1908971250108*cos(0.8184*t*78))+(4.24654940944126*sin(0.8184*t*78)))+
((-10.8191993290903*cos(0.8184*t*79))+(2.92947907217917*sin(0.8184*t*79)))+
((-11.5454027505307*cos(0.8184*t*80))+(3.26446738294726*sin(0.8184*t*80)))+
((-10.3483423584431*cos(0.8184*t*81))+(3.1160454389992*sin(0.8184*t*81)))+
((-9.70029651689084*cos(0.8184*t*82))+(3.54492320128412*sin(0.8184*t*82)))+
((-8.73135195461731*cos(0.8184*t*83))+(0.590857390868776*sin(0.8184*t*83)))+
((-8.94847362919554*cos(0.8184*t*84))+(1.32793130131655*sin(0.8184*t*84)))+
((-6.43005687516546*cos(0.8184*t*85))+(-1.0303702908659*sin(0.8184*t*85)))+
((1.22143209941039*cos(0.8184*t*86))+(-4.92770962318511*sin(0.8184*t*86)))+
((-35.8523216210297*cos(0.8184*t*87))+(16.168234110332*sin(0.8184*t*87)))+
((-19.389656070412*cos(0.8184*t*88))+(6.11556589909943*sin(0.8184*t*88)))+
((-16.8885352843545*cos(0.8184*t*89))+(3.15928700144396*sin(0.8184*t*89)))+
((-16.8966575151449*cos(0.8184*t*90))+(3.3570498188947*sin(0.8184*t*90)))+

```
((-15.6269882458775*cos(0.8184*t*91))+(3.26997033263291*sin(0.8184*t*91)))+
((-15.3348084311456*cos(0.8184*t*92))+(1.26031790968583*sin(0.8184*t*92)))+
((-16.6107194008582*cos(0.8184*t*93))+(0.795860897472321*sin(0.8184*t*93)))+
((-18.465324928285*cos(0.8184*t*94))+(-2.21219304363687*sin(0.8184*t*94)))+
((-25.0935555361424*cos(0.8184*t*95))+(-6.21409961995961*sin(0.8184*t*95)))+
((-73.2543784009361*cos(0.8184*t*96))+(-44.643786646671*sin(0.8184*t*96)))+
((10.8213258716677*cos(0.8184*t*97))+(20.4587859458835*sin(0.8184*t*97)))+
((-1.54400076307518*cos(0.8184*t*98))+(10.6893007486501*sin(0.8184*t*98)))+
((-4.74814169912527*cos(0.8184*t*99))+(8.39797288564973*sin(0.8184*t*99)))+
((-5.64152896900597*cos(0.8184*t*100))+(6.93971091858896*sin(0.8184*t*100)))+
((-6.64759656885455*cos(0.8184*t*101))+(5.58528409802487*sin(0.8184*t*101)))+
((-9.06702128541373*cos(0.8184*t*102))+(4.26213733174568*sin(0.8184*t*102)))+
((-8.03475966295462*cos(0.8184*t*103))+(4.99712560656189*sin(0.8184*t*103)))+
((-8.83478405918337*cos(0.8184*t*104))+(3.41362467384176*sin(0.8184*t*104)))+
((-8.16861617216769*cos(0.8184*t*105))+(3.82287366430839*sin(0.8184*t*105)))+
((-12.7214051387143*cos(0.8184*t*106))+(21.0609431799553*sin(0.8184*t*106)))+
((-9.86904647914842*cos(0.8184*t*107))+(6.59608096145454*sin(0.8184*t*107)))+
((-10.1337366373202*cos(0.8184*t*108))+(5.55301705757618*sin(0.8184*t*108)))+
((-9.81027580094264*cos(0.8184*t*109))+(6.04241357200281*sin(0.8184*t*109)))+
((-9.22736172066468*cos(0.8184*t*110))+(5.48292172711953*sin(0.8184*t*110)))+
((-10.4963127463151*cos(0.8184*t*111))+(4.68344802442455*sin(0.8184*t*111)))+
((-9.57833835965702*cos(0.8184*t*112))+(6.03759098992706*sin(0.8184*t*112)))+
((-10.4734647534639*cos(0.8184*t*113))+(6.72026250180016*sin(0.8184*t*113)))+
((-11.4743184261589*cos(0.8184*t*114))+(9.9209786832631*sin(0.8184*t*114)))+
((-14.0571738970304*cos(0.8184*t*115))+(21.4935591046248*sin(0.8184*t*115)))+
((-5.32751414106385*cos(0.8184*t*116))+(-16.9445501931388*sin(0.8184*t*116)))+
((-7.98220076435859*cos(0.8184*t*117))+(-3.86449436966366*sin(0.8184*t*117)))+
((-7.80426569391827*cos(0.8184*t*118))+(-1.73476784551681*sin(0.8184*t*118)))+
((-8.89347554201169*cos(0.8184*t*119))+(-0.948413404126034*sin(0.8184*t*119)))+
((-9.07542005013518*cos(0.8184*t*120))+(-0.033714888526718*sin(0.8184*t*120)))+
((-10.0999399159378*cos(0.8184*t*121))+(2.33093570213312*sin(0.8184*t*121)))+
((-8.35193903261376*cos(0.8184*t*122))+(0.584858385805653*sin(0.8184*t*122)))+
((-9.13990155997818*cos(0.8184*t*123))+(0.815827466773143*sin(0.8184*t*123)))+
((-7.45250099962571*cos(0.8184*t*124))+(0.906538145433625*sin(0.8184*t*124)))+
((-3.80423305047201*cos(0.8184*t*125))+(-2.86629861790881*sin(0.8184*t*125)));
}
 end_f_loop(f,thread)
}
```
